# Socio-Economic Position Has No Effect on Improvement in Health-Related Quality of Life and Patient Satisfaction in Total Hip and Knee Replacement: A Cohort Study

**DOI:** 10.1371/journal.pone.0056785

**Published:** 2013-03-08

**Authors:** J. Christiaan Keurentjes, David Blane, Melanie Bartley, Johan J. B. Keurentjes, Marta Fiocco, Rob G. Nelissen

**Affiliations:** 1 Department of Orthopaedic Surgery, Leiden University Medical Center, Leiden, The Netherlands; 2 Department of Primary Care and Public Health, Imperial College London, London, United Kingdom; 3 Department of Epidemiology and Public Health, University College London, London, United Kingdom; 4 Department of Information Management, Kadaster/Dutch Land Registry Office, Apeldoorn, The Netherlands; 5 Department of Medical Statistics and BioInformatics, Leiden University Medical Center, Leiden, The Netherlands; Cochin Hospital (AP-HP), and the University Paris Descarte, France

## Abstract

**Introduction:**

Considerable evidence suggests that patients with more advantaged Socio-Economic Positions undergo Total Hip and Knee Replacement (THR/TKR) more often, despite having a lower need. We questioned whether more disadvantaged Socio-Economic Position is associated with an lower improvement in Health-Related Quality of Life (HRQoL) and a lower patient satisfaction after THR/TKR.

**Methods:**

Patients who underwent primary THR/TKR in one academic and three community hospitals between 2005 and 2009, were eligible for inclusion. The highest completed levels of schooling were aggregated to index social class. We compared the improvement in HRQoL and postoperative satisfaction with surgery (measured using the Short-Form 36 (SF36) and an 11-point numeric rating scale of satisfaction) between the aggregated groups of highest completed levels of schooling, using linear mixed model analysis, with center as a random effect and potential confounders (i.e. age, gender, Body Mass Index and Charnley's comorbidity classification) as fixed effects.

**Results:**

586 THR patients and 400 TKR patients (40% of all eligible patients) agreed to participate and completed all questionnaires sufficiently. We found no differences in HRQoL improvement in any dimension of the SF36 in THR patients. Patients with a higher completed level of schooling had a larger improvement in role-physical (9.38 points, 95%-CI:0.34–18.4), a larger improvement in general health (3.67 points, 95%-CI:0.56–6.79) and a smaller improvement in mental health (3.60 points, 95%-CI:0.82–6.38) after TKR. Postoperative patient satisfaction did not differ between different highest completed level of schooling groups.

**Discussion:**

Completed level of schooling has no effect on the improvement in HRQoL and patient satisfaction in a Dutch THR population and a small effect in a similar TKR population. Undertreatment of patients with more disadvantaged Socio-Economic Position cannot be justified, given the similar improvement in HRQoL and postoperative level of satisfaction with surgery between the social groups examined.

## Introduction

Total Hip Replacement (THR) and Total Knee Replacement (TKR) are effective surgical interventions, which alleviate pain and improve Health-Related Quality of Life (HRQoL) in patients with hip or knee joint degeneration.[1] Although on average patients improve markedly after THR or TKR, not all patients benefit from these surgeries. Persistent pain is reported in 9% of THR patients and 20% of TKR patients at long term follow-up.[2] Additionally, up to 30% of patients are dissatisfied after surgery, with higher reported dissatisfaction rates for TKR patients.[3]–[9] Therapeutic options are limited in patients with persistent pain or dissatisfaction after joint replacement: the outcome of revision surgery performed without a specific mechanical or physiological indication is highly unpredictable. Furthermore, revision THR or TKR surgery is associated with a higher probability of orthopaedic and medical complications. Given the projected increase of 137% and 601% in the annual number of THR and TKR performed in the United States in 2030, the absolute number of patients with unfavorable outcomes after joint replacement is expected to rise, potentially inducing large societal and medical problems.[10]

Predicting which patient groups are at increased risk of an unfavorable outcome after joint replacement may provide additional insights in the mechanisms involved and offer the possibility of intervention in order to optimise the outcome. At the very least, it allows patients to be well informed of their specific risks and expected gains before surgery.

People attain unequal societal positions according to their occupation, educational achievement, income level and status. The Socio-Economic Position (SEP) encompasses both resource-based measures and prestige-based measures in determining an individuals position in the socioeconomic hierarchy.[11] The patients SEP might be a good predictor of a favorable outcome after joint replacement: a more advantaged SEP is associated with better health,[12] which in turn is associated with better outcomes after joint replacement surgery.[13], [14] As it does not require any invasive or expensive diagnostics, it would be easy to implement in clinical practice. We therefore questioned whether SEP was associated with the improvement in HRQoL and satisfaction after THR or TKR. We hypothesised that patients with more advantaged SEP would have a larger improvement in HRQoL after THR and TKR and a higher degree of satisfaction with their surgical results.

## Methods

**Figure 1:**
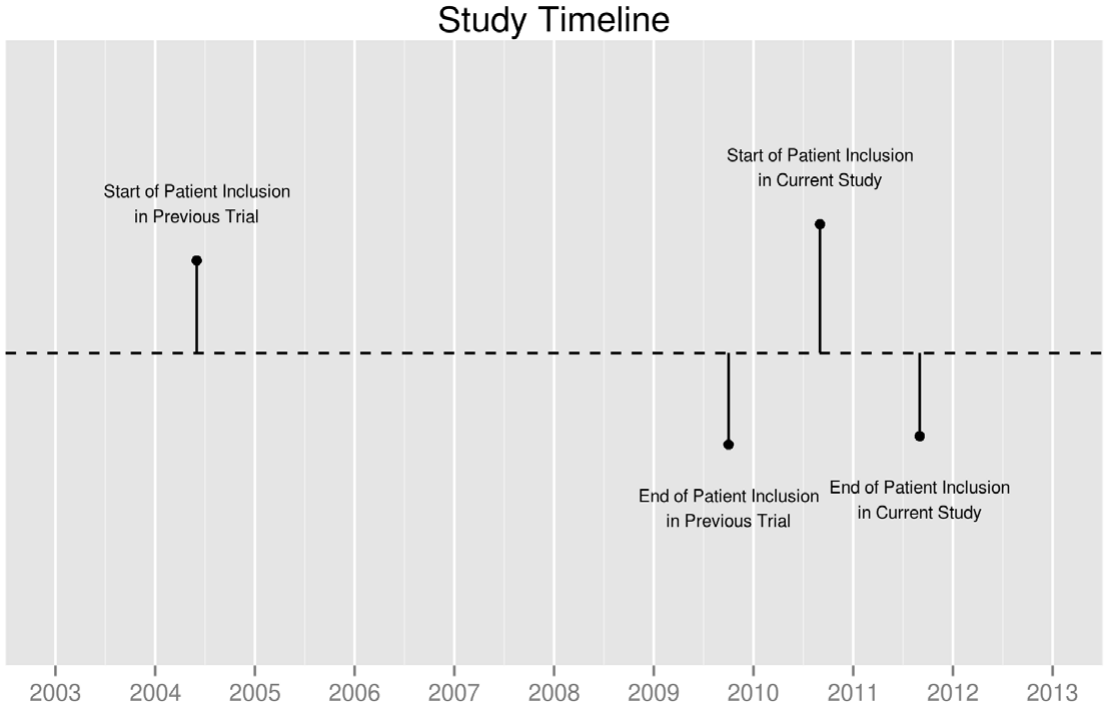
Study Timeline.

The presently reported study is an add-on to a multi-center follow-up study, conducted at the departments of orthopaedic surgery of the Leiden University Medical Center, the Slotervaart hospital in Amsterdam, the Albert Schweitzer hospital in Dordrecht and the Groene Hart hospital in Gouda, the Netherlands, from August 2010 until August 2011 (see Study Time-line in figure 1). The study was approved by the Medical Ethics Committee of the Leiden University Medical Center and the Medical Ethical Committees of all other participating centers; all patients gave written informed consent (CCMO-Nr: NL29018.058.09; MEC-Nr: P09.189). This study was registered in the Netherlands Trial Register (NTR2190). It concerned the clinical follow-up of a multi-center randomized controlled clinical trial, comparing different blood management modalities in THR and TKR surgery (Netherlands Trial Register: NTR303). In this trial, 2442 primary and revision hip or knee replacements in 2257 patients were included between 2004 and 2009 (see Study Time-line in figure 1).

All patients who participated in the randomized controlled trial and completed preoperative HRQoL questionnaires, who underwent primary THR of TKR and who were alive at the time of inclusion for the present follow-up study were eligible for inclusion. In contrast to the previous clinical trial, in which joint replacements were the subjects of interest, patients are the subject of interest in the current study. Patients who participated more than once in the previous trial, were only allowed to participate once in the current study; the first joint replacement performed in the previous trial was chosen as the index surgery.

Records of the financial administration of all participating centers were checked in order to ascertain that all eligible patients were still alive before being approached. All eligible patients were first sent an invitation letter signed by their treating orthopaedic surgeon, an information brochure and a reply card. Patients who did not respond within 4 weeks after the first invitation were sent another invitation letter. The remaining patients, who did not respond to this second invitation, were contacted by telephone.

The improvement in different dimensions of HRQoL and satisfaction with the surgical results were the outcome measures of interest. Important concepts in HRQoL are elements of health status that people usually value (e.g. stair climbing) and peoples rating of the value of their subjective experience of living.[15] In other words, both objective functioning and subjective well-being should be considered when measuring HRQoL.[16] We measured HRQoL preoperatively and in the present follow-up study using the Short-Form 36 (SF36),[17] a health status instrument which includes several sub-scales related to functioning as well as perceived well-being.[18], [19] The SF36 is translated and validated in Dutch and allows studying small between-group differences in HRQoL.[20], [21] The 36 items cover eight domains (physical function, role physical, bodily pain, general health, vitality, social function, role emotional, and mental health), for which a sub-scale score is calculated (100 indicating no symptoms and 0 indicating extreme symptoms). Additionally, these scales are incorporated into two summary measures: a Physical Component Summary (PCS) and Mental Component Summary (MCS). The HRQoL outcome measure was the mean improvement (i.e. the mean of each patients postoperative sub-scale score minus their preoperative sub-scale score).

At follow-up one question was asked about satisfaction with the result in general, namely: “How satisfied are you with your hip or knee replacement?”. Such as a single item has been shown to provide additional insight into the impact of surgery, besides the measurement of HRQoL.[22] Patient satisfaction with the surgical result was measured using an 11-point Numeric Rating Scale of Satisfaction (NRSS; 0 indicating completely dissatisfied, 10 indicating completely satisfied). The satisfaction outcome measure was the mean NRSS score.

### Exposure

The follow-up questionnaire contained the following question: “What is your highest completed level of schooling?”. We have aggregated these levels of schooling into an approximation of the social classes, on the assumption that level of schooling indexes the type of qualifications obtained, which in turn indicates the type of occupations available to the subject and hence their own adult social class. Thus: University, Higher vocational education and Preparatory higher vocational & scientific education have been aggregated as indicating the professional and managerial social classes; Middle vocational education and Preparatory middle vocational education have been aggregated as indicating the skilled non-manual and manual social classes; and Lower vocational education, Elementary schooling and No formal education have been aggregated as indicating the semi- & unskilled manual social classes.

### Potential confounders

Sociodemographic characteristics collected at baseline in the trial included: age at joint replacement and gender. Additionally, the following variables were collected in the questionnaire of the follow-up study: length and weight, in order to calculate the Body Mass Index (BMI) (<25, 25–30, 30–35, >35) and patient reported Charnley classification of co-morbidity (Class A: patients in which the index operated hip or knee are affected only; Class B: patients in which the other hip or knee is affected as well; Class C: patients with a hip or knee replacement and other affected joints and/or a medical condition which affects the patients' ability to ambulate).[23], [24]

### Statistical analysis

We performed descriptive analyses of patients baseline characteristics. In order to investigate the possible extent of self-selection bias, we compared the age at THR or TKR and gender of participants to non-participants.

Patients with missing preoperative SF36 questionnaires, missing SF36 questionnaires at follow-up or missing highest level of schooling were excluded from analyses, as we could not exclude a Missing Not At Random (MNAR) mechanism. Missing values of the Charnley Co-morbidity Classification and BMI were deemed Missing At Random and imputed using Multiple Imputations (MI), in order to improve efficiency of the regression analyses and avert biased regression coefficients. We performed MI (m = 10) using an Expectation-Maximization algorithm,[25] which is implemented in the Amelia 2 package for R.[26], [27]

We performed regression analyses in each imputed dataset in order to compare the mean improvement in HRQoL and the mean NRSS between patients from different social classes, whilst adjusting for confounders. As minimal clinically important differences (MCIDs) in HRQoL differ between THR patients and TKR patients,[28] we performed all analyses separately for THR and TKR. Possible confounders are age, gender, BMI and poly-articular morbidity in both THR and TKR patients. We used the Charnley classification as a proxy for poly-articular morbidity. As the length of follow-up varies considerably, we first stratified our data in quartiles of follow-up length for each imputed dataset. Within each stratum of follow-up length, we performed a multivariate mixed effect linear regression analysis, with the mean improvement in HRQoL and the mean NRSS as the dependent variable, the completed level of schooling and confounders as independent variables and center as a random effect. Stratum-specific mean differences in HRQoL between the KL grades were pooled using inverse variance weighting in order to produce an overall estimate of the mean difference in HRQoL for each imputed data-set. Finally, the m = 10 estimates of the mean differences in HRQoL were combined into one estimate, according to Rubin.[29]

All analyses were performed using R, version 2.14.0.[30]

## Results

In the previous trial, 2579 THR and TKR were randomised in 2382 patients; 2442 joint replacements were evaluated. The first joint replacements of the 2382 patients consisted of 2206 primary THR and TKR and 176 revision THR and TKR. Of these 2206 patients who underwent primary joint replacement, 285 patients did not complete all preoperative questionnaires and 63 patients died, leaving 1858 patients with primary joint replacement eligible. 986 patients agreed to participate, of which 668 patients had returned all questionnaires sufficiently completed (response rate: 40%). Non-responding THR patients were on average 3.95 years older than participants (95%CI: 2.6–5.3 years); Non-responding TKR patients were on average 3.31 years older than participants (95%CI: 2.0–4.7 years). The proportion of males was similar in participants and non-responders. An overview of the patient characteristics is provided in table 1, an overview of preoperative HRQoL is presented in table 2 for THR patients and 3 for TKR patients.

**Table 1:** Patient Characteristics.

HIP	University, Higher Vocational Education and Preparatory Higher Vocational & Scientific Education: n = 100	Middle Vocational Education and Preparatory Middle Vocational Education n = 150	Lower Vocational Education, Elementary Schooling and No Formal Education n = 156	All Patients: n = 406
Age	62.5 (11.9)	63.8 (10.6)	66.3 (9.4)	64.4 (10.6)
Males	51.0%	34.7%	35.9%	39.2%
Follow-up period	3.13 (1.20)	3.19 (1.10)	3.17 (1.10)	3.16 (1.14)
Charnley:				
A	24.2%	20.0%	22.6%	22.0%
B	12.6%	17.9%	11.6%	14.2%
C	63.2%	62.1%	65.8%	63.7%
BMI:				
<25	50.0%	33.8%	27.0%	35.0%
25–30	35.1%	41.2%	48.0%	42.4%
30–35	12.8%	20.3%	19.1%	18.0%
>35	2.10%	4.70%	5.90%	4.60%
**KNEE**	**n = 42**	**n = 98**	**n = 122**	**n = 262**
Age	63.7 (12.7)	67.6 (9.0)	69.2 (9.3)	67.7 (10.0)
Males	40.5%	35.7%	29.5%	33.6%
Follow-up period	3.25 (1.2)	3.02 (1.0)	3.28 (1.2)	3.18 (1.13)
Charnley:				
A	14.6%	14.0%	11.7%	13.0%
B	14.6%	10.8%	10.0%	11.0%
C	70.6%	75.3%	78.3%	76.0%
BMI:				
<25	23.7%	13.7%	15.5%	16.1%
25–30	47.4%	56.8%	37.9%	46.6%
30–35	23.7%	21.1%	26.7%	24.1%
>35	5.30%	8.40%	19.8%	13.3%

Values are means (SD), unless stated otherwise.

**Table 2:** Quality of Life before Hip Replacement: A Comparison Between Patients with different Completed Levels of Schooling.

SF36 Sub-Scale	University, Higher Vocational Education and Preparatory Higher Vocational & Scientific Education: n = 100	Middle Vocational Education and Preparatory Middle Vocational Education n = 150	Lower Vocational Education, Elementary Schooling and No Formal Education n = 156	All Patients: n = 406
Physical Functioning	43.0 (20.2)	39.1 (21.7)	39.8 (22.5)	40.3 (21.6)
Role-Physical	38.4 (40.7)	31.7 (39.6)	28.6 (38.1)	32.2 (39.4)
Bodily Pain	44.3 (19.3)	41.7 (20.6)	38.4 (20.7)	41.1 (20.4)
General Health	70.0 (19.9)	69.1 (19.4)	67.6 (19.3)	68.7 (19.5)
Vitality	67.2 (20.7)	59.6 (20.6)	59.5 (22.8)	61.4 (21.7)
Social Functioning	69.0 (22.8)	66.2 (26.6)	63.8 (30.6)	66.0 (27.4)
Role Emotional	79.7 (36.4)	71.1 (41.7)	67.1 (41.7)	71.7 (40.6)
Mental Health	78.9 (15.8)	74.0 (18.1)	73.4 (19.6)	75.0 (18.3)
PCS	38.0 (11.1)	38.5 (9.10)	38.8 (9.40)	38.5 (9.70)
MCS	54.8 (9.30)	51.7 (10.9)	50.9 (11.1)	52.2 (10.7)

Values are means (SD).

Data on age, gender, highest completed level of schooling, pre- and postoperative SF36, satisfaction with surgery and length of follow-up was complete for all THR patients and all TKR patients. In 20 THR patients and 8 TKR patients, the Charnley classification was missing; in 12 THR patients and 13 TKR patients, the BMI was missing.

The mean improvement in HRQoL and mean NRSS per completed level of schooling is shown in table 4 for THR patients and table 5 for TKR patients. Adjusted differences in improvement in HRQoL and mean NRSS after joint replacement per increasing category of completed level of schooling are shown in table 6 for THR patients and table 7 for TKR patients. For each increasing completed level of schooling, THR patients improved 0.88 points more in physical functioning, 3.09 points less in role-physical, 0.60 points less in bodily pain, 0.66 points less in general health, 1.44 points less in vitality, 0.12 points more in social functioning, 0.34 points less in role-emotional, 1.35 points less in mental health, 0.17 points less in the physical component summary and 0.80 points less in the mental component summary; however, none of these differences reached statistical significance (table 6). For each increasing completed level of schooling, TKR patients improved 3.64 points more in physical functioning, 9.38 points more in role-physical, 3.68 points more in bodily pain, 3.67 points more in general health, 1.78 points less in vitality, 0.62 points more in social functioning, 3.11 points less in role-emotional, 3.60 points less in mental health, 2.74 points more in the physical component summary and 2.08 points less in the mental component summary; however, only role-physical, general health, mental health, the physical component summary and the mental component summary reached statistical significance (table 7).

**Table 4:** Improvement in Health-Related Quality of Life and Satisfaction after Hip Replacement:: A Comparison Between Patients with different Completed Levels of Schooling.

SF36 Sub-Scale	University, Higher Vocational Education and Preparatory Higher Vocational & Scientific Education: n = 100	Middle Vocational Education and Preparatory Middle Vocational Education n = 150	Lower Vocational Education, Elementary Schooling and No Formal Education n = 156
Physical Functioning	27.8 (23.3–32.3)	26.6 (22.4–30.7)	24.9 (20.5–29.2)
Role-Physical	35.7 (26.6–44.7)	40.7 (32.9–48.6)	42.3 (35.0–49.5)
Bodily Pain	38.0 (33.1–42.9)	33.4 (29.3–37.6)	38.9 (34.9–42.9)
General Health	−1.20 (−4.80–2.50)	−0.70 (−3.70–2.30)	−0.20 (−3.50–3.10)
Vitality	3.40 (0.20–6.60)	8.50 (5.80–11.3)	6.70 (3.10–10.2)
Social Functioning	16.0 (11.3–20.7)	18.1 (13.5–22.7)	20.0 (15.4–24.5)
Role Emotional	5.70 (−2.60–13.9)	16.0 (8.80–23.2)	11.9 (4.70–19.0)
Mental Health	2.10 (−0.60–4.80)	6.40 (3.90–9.00)	5.90 (3.30–8.50)
PCS	12.8 (11.1–14.6)	10.8 (9.20–12.3)	11.4 (9.80–13.0)
MCS	−1.60 (−3.40–0.30)	1.50 (−0.10–3.00)	0.60 (−1.00–2.20)
NRS Satisfaction	8.9 (8.6–9.3)	8.7 (8.4–9.0)	8.6 (8.3–8.9)

**Table 5:** Improvement in Health-Related Quality of Life and Satisfaction after Knee Replacement:: A Comparison Between Patients with different Completed Levels of Schooling.

SF36 Sub-Scale	University, Higher Vocational Education and Preparatory Higher Vocational & Scientific Education: n = 42	Middle Vocational Education and Preparatory Middle Vocational Education n = 98	Lower Vocational Education, Elementary Schooling and No Formal Education n = 122
Physical Functioning	20.4 (12.4–28.5)	14.0 (8.70–19.3)	10.3 (6.40–14.2)
Role-Physical	31.5 (15.6–47.5)	25.2 (14.4–35.9)	15.6 (7.40–23.7)
Bodily Pain	24.9 (17.6–32.2)	25.5 (19.7–31.2)	21.0 (16.5–25.5)
General Health	4.00 (−0.60–8.60)	−1.60 (−5.20–2.10)	−3.60 (−6.90–−0.30)
Vitality	1.30 (−3.60–6.30)	−1.00 (−4.70–2.80)	2.60 (−0.90–6.10)
Social Functioning	11.6 (2.80–20.4)	7.80 (1.80–13.8)	8.80 (4.40–13.2)
Role Emotional	9.50 (−1.50–20.5)	3.40 (−6.30–13.1)	10.9 (2.60–19.2)
Mental Health	3.30 (0.20–6.40)	1.30 (−2.30–4.90)	6.20 (3.40–9.10)
PCS	7.70 (4.20–11.3)	7.00 (4.90–9.00)	4.20 (2.70–5.70)
MCS	−0.40 (−3.00–2.20)	−1.70 (−3.7–0.30)	1.70 (0.00–3.40)
NRS Satisfaction	8.3 (7.6–9.1)	8.1 (7.6–8.6)	7.9 (7.4–8.4)

**Table 6:** Adjusted Difference in Improvement in Health-Related Quality of Life and Satisfaction after Hip Replacement: A Comparison Between Patients with different Completed Levels of Schooling.

SF36 Sub-Scale	Adjusted difference per increasing Completed Levels of Schooling (95% CI)	p-value
Physical Functioning	−0.88 (−4.14–2.38)	0.59
Role-Physical	3.09 (−2.89–9.07)	0.31
Bodily Pain	0.60 (−2.70–3.89)	0.72
General Health	0.66 (−1.81–3.13)	0.60
Vitality	1.44 (−1.04–3.92)	0.25
Social Functioning	−0.12 (−3.59–3.36)	0.94
Role Emotional	0.34 (−5.31–6.00)	0.90
Mental Health	1.35 (−0.61–3.30)	0.18
PCS	0.17 (−1.04–1.38)	0.79
MCS	0.80 (−0.42–2.03)	0.20
NRS Satisfaction	−0.1 (−0.4–0.1)	0.29

Negative values indicate a higher mean improvement in HRQoL after THR in patients with increasing Completed Levels of Schooling. The mean differences between education level are adjusted for age, sex, Body Mass Index and Charnley Classification of Comorbidity and stratified for quartiles of follow-up.

**Table 7:** Adjusted Difference in Improvement in Health-Related Quality of Life and Satisfaction after Knee Replacement: A Comparison Between Patients with different Completed Levels of Schooling.

SF36 Sub-Scale	Adjusted difference per increasing Completed Levels of Schooling (95% CI)	p-value
Physical Functioning	−3.64 (−8.03–0.74)	0.10
Role-Physical	−9.38 (−18.4–−0.34)	0.04
Bodily Pain	−3.68 (−8.39–1.03)	0.13
General Health	−3.67 (−6.79–−0.56)	0.02
Vitality	1.78 (−1.51–5.08)	0.29
Social Functioning	−0.62 (−5.37–4.14)	0.80
Role Emotional	3.11 (−5.07–11.3)	0.46
Mental Health	3.60 (0.82–6.38)	0.01
PCS	−2.74 (−4.41–−1.07)	0.001
MCS	2.08 (0.37–3.79)	0.02
NRS Satisfaction	0.0 (−0.5–0.4)	0.83

Negative values indicate a higher mean improvement in HRQoL after TKR in patients with increasing Completed Levels of Schooling. The mean differences between education level are adjusted for age, sex, Body Mass Index and Charnley Classification of Comorbidity and stratified for quartiles of follow-up.

For each increasing completed level of schooling, the NRSS increased 0.1 points for THR patients and 0.0 points for TKR patients. None of these differences reached statistical significance (table 6 and 7).

Adjusted differences in improvement in HRQoL and mean NRSS after joint replacement between each completed level of schooling category are shown in table 8 for THR patients and table 9 for TKR patients. The larger improvement in role-physical functioning in patients with a higher level of completed schooling is mainly due to the large difference between patients with Middle Vocational Education or Preparatory Middle Vocational Education and patients with Lower Vocational Education, Elementary Schooling or No Formal Education. The larger improvement in general health is constant across all groups of level of completed schooling. The larger improvement in the Physical Component Summary Scale in patients with a higher level of completed schooling is mainly due to the large difference between patients with Middle Vocational Education or Preparatory Middle Vocational Education and patients with Lower Vocational Education, Elementary Schooling or No Formal Education.

**Table 8:** Adjusted Difference in Improvement in Health-Related Quality of Life and Satisfaction after Hip Replacement.: A Comparison Between Patients with different Completed Levels of Schooling.

SF36 Sub-Scale	Adjusted Difference Between U+HVE+PHVSE and MVE+PMVE* (95%CI)	Adjusted Difference Between MVE+PMVE and LVE+ES+NFE** (95%CI)	Adjusted Difference Between U+HVE+PHVSE and LVE+ES+NFE*** (95%CI)
Physical Functioning	−1.96 (−8.56–4.64)	0.91 (−5.16–6.97)	−1.94 (−8.53–4.66)
Role-Physical	6.21 (−5.85–18.3)	2.66 (−8.04–13.4)	6.21 (−5.91–18.3)
Bodily Pain	−6.27 (−12.9–0.33)	7.28 (1.69–12.7)	−0.33 (−6.94–6.29)
General Health	−0.70 (−5.72–4.31)	1.57 (−2.95–6.08)	1.16 (−3.85–6.17)
Vitality	5.11 (0.09–10.1)	−1.08 (−5.76–3.61)	3.18 (−1.83–8.19)
Social Functioning	−0.50 (−7.57–6.57)	0.85 (−5.72–7.42)	−0.34 (−7.37–6.70)
Role Emotional	5.63 (−5.81–17.1)	−2.95 (−13.3–7.42)	1.89 (−9.59–13.4)
Mental Health	3.43 (−0.59–7.44)	−0.19 (−3.86–3.48)	2.98 (−0.99–6.95)
PCS	−1.64 (−4.07–0.79)	1.85 (−0.29–3.99)	−0.05 (−2.49–2.38)
MCS	2.69 (0.20–5.19)	−0.70 (−2.95–1.55)	1.93 (−0.55–4.41)
NRS Satisfaction	−0.3 (−0.8–0.2)	0.1 (−0.3–0.6)	−0.3 (−0.9–0.2)

U+HVE+PHVSE: University, Higher Vocational Education and Preparatory Higher Vocational & Scientific Education; MVE+PMVE: Middle Vocational Education and Preparatory Middle Vocational Education; LVE+ES+NFE: Lower Vocational Education, Elementary Schooling and No Formal Education.

* Negative values indicate a higher mean improvement in HRQoL after THR in U+HVE+PHVSE patients, compared to MVE+PMVE patients.

** Negative values indicate a higher mean improvement in HRQoL after THR in MVE+PMVE patients, compared to LVE+ES+NFE patients.

*** Negative values indicate a higher mean improvement in HRQoL after THR in U+HVE+PHVSE patients, compared to LVE+ES+NFE patients.

The mean differences between education level are adjusted for age, sex, Body Mass Index and Charnley Classification of Comorbidity and stratified for quartiles of follow-up.

**Table 9:** Adjusted Difference in Improvement in Health-Related Quality of Life and Satisfaction after Knee Replacement::A Comparison Between Patients with different Completed Levels of Schooling.

SF36 Sub-Scale	Adjusted Difference Between U+HVE+PHVSE and MVE+PMVE* (95%CI)	Adjusted Difference Between MVE+PMVE and LVE+ES+NFE** (95%CI)	Adjusted Difference Between U+HVE+PHVSE and LVE+ES+NFE*** (95%CI)
Physical Functioning	−5.80 (−15.1–3.53)	−2.24 (−9.14–4.65)	−7.99 (−17.2–1.26)
Role-Physical	−3.37 (−22.5–15.7)	−12.2 (−26.5–2.18)	−16.5 (−35.4–2.53)
Bodily Pain	1.00 (−9.02–11.0)	−6.46 (−14.1–1.20)	−6.13 (−16.1–3.81)
General Health	−4.84 (−11.4–1.72)	−2.84 (−7.88–2.21)	−7.64 (−14.2–−1.10)
Vitality	−0.60 (−7.57–6.36)	3.57 (−1.70–8.83)	2.98 (−3.93–9.89)
Social Functioning	−3.22 (−13.4–6.94)	0.87 (−6.47–8.21)	−2.09 (−12.2–7.94)
Role Emotional	−1.26 (−18.8–16.2)	5.73 (−7.79–19.3)	4.49 (−12.8–21.8)
Mental Health	−0.80 (−6.69–5.08)	5.84 (1.11–10.6)	6.32 (0.53–12.1)
PCS	−1.36 (−4.90–2.18)	−3.42 (−6.04–−0.80)	−5.04 (−8.56–−1.52)
MCS	0.03 (−3.63–3.70)	3.04 (0.19–5.88)	3.58 (−0.03–7.19)
NRS Satisfaction	0.2 (−0.8–1.1)	−0.1 (−0.8–0.6)	−0.1 (−1.0–0.8)

U+HVE+PHVSE: University, Higher Vocational Education and Preparatory Higher Vocational & Scientific Education; MVE+PMVE: Middle Vocational Education and Preparatory Middle Vocational Education; LVE+ES+NFE: Lower Vocational Education, Elementary Schooling and No Formal Education.

* Negative values indicate a higher mean improvement in HRQoL after TKR in U+HVE+PHVSE patients, compared to MVE+PMVE patients.

** Negative values indicate a higher mean improvement in HRQoL after TKR in MVE+PMVE patients, compared to LVE+ES+NFE patients.

*** Negative values indicate a higher mean improvement in HRQoL after TKR in U+HVE+PHVSE patients, compared to LVE+ES+NFE patients.

The mean differences between education level are adjusted for age, sex, Body Mass Index and Charnley Classification of Comorbidity and stratified for quartiles of follow-up.

The smaller improvement in mental health in patients with a higher level of completed schooling is mainly due to the large difference between patients with Middle Vocational Education or Preparatory Middle Vocational Education and patients with Lower Vocational Education, Elementary Schooling or No Formal Education.

Finally, the smaller improvement in the Mental Component Summary Scale in patients with a higher level of completed schooling is mainly due to the large difference between patients with Middle Vocational Education or Preparatory Middle Vocational Education and patients with Lower Vocational Education, Elementary Schooling or No Formal Education.

## Discussion

Regardless of their completed level of schooling, patients improve in HRQoL and have a high satisfaction after THR. After TKR, we found that patients with higher completed levels of schooling had a larger improvement in role-physical functioning, general health and the Physical Component Summary scale and a smaller improvement in mental health and the Mental Component Summary scale, although the found differences in the SF36 subscales were smaller than recently published within-group MCIDs at two-years follow-up.[28] All other dimensions of HRQoL and patient satisfaction showed no differences between the completed levels of schooling, thereby failing to refute our hypothesis.

Strengths of our study include the rigorous efforts to minimise confounding and the generalisability of our study population, due to the multi-center setting and the similarity of the demographics of our study population to those of large-scaled national joint registries.[31]

Weaknesses of the study include the low participation rate and the variation in follow-up period after joint replacement. Although participation rates of 100% are feasible in small-scaled studies with hard endpoints,[32], [33] participation rates in epidemiological studies have been steadily declining in the last 30 years.[34] Even sharper declines have been reported in the past few years.[35] Unfortunately, the participation rate of this study follows this general trend, and therefore we cannot exclude the presence of self-selection bias. In order to limit the extent of this bias, we have sent multiple reminders and have called all patients who did not answer our reminders and who did not return the questionnaire. As incentives, we have included an appealing information brochure in which the primary goals of the follow-up study were explained and a study pen as a small gift. Additionally, patients were urged to participate by their treating physician. However, the participation rate alone does not determine the extent of bias present in any particular study.[35] The difference between participants and nonparticipants is far more important.[36] As the found differences in demographics were small, it is unlikely that the study results will be severely biased.

**Figure 2:**
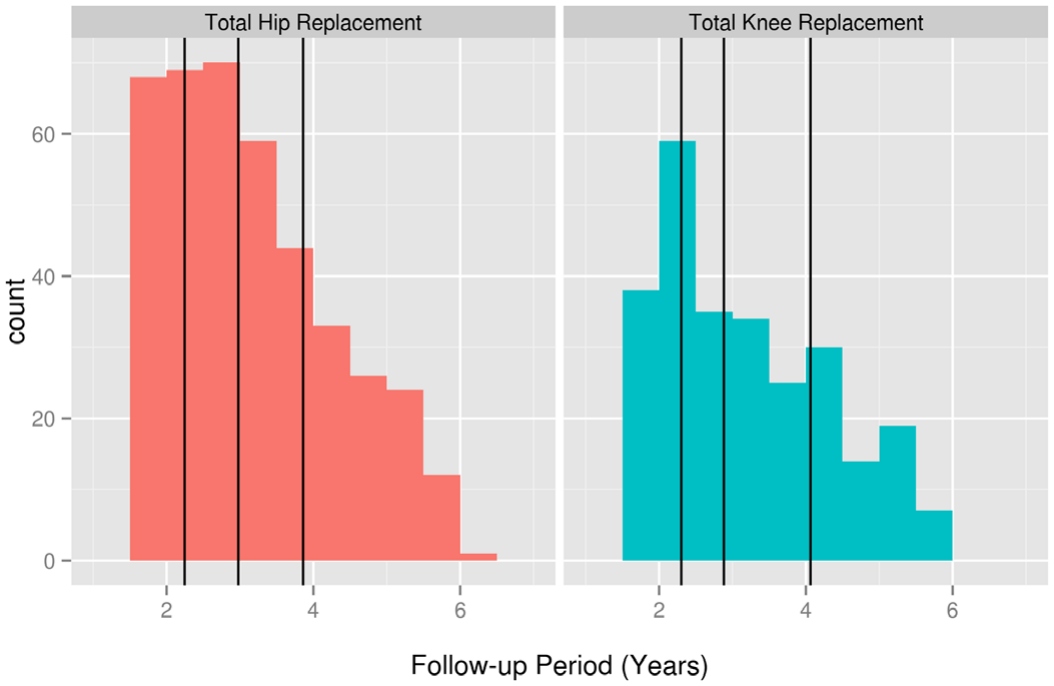
Follow-up Period in Years for THR and TKR patients. Vertical lines indicate quartiles of follow-up period length.

The follow-up period after joint replacement varies between 1.5 and 6 years in this study (Figure 1 and 2). Theoretically, this broad range could influence our findings. In order to exclude this variable, all patients should have been followed for the exact same amount of time. In our data, we found no clear evidence of a relationship between the improvement in HRQoL after joint replacement and the follow-up period (See Appendix S1 and S2 for scatter plots of the improvement in HRQoL as a function of the follow-up period length, stratified per completed levels of schooling and Appendix S3 and S4 for scatter plots of the NRSS after surgery as a function of the follow-up period length, stratified per completed levels of schooling). In order to account for this range, we stratified our analysis per quartile of follow-up period. Stratifying for an additional variable inevitably leads to a loss of power, thereby increasing the probability of a type 2-error. In our analysis, this loss of power was negligible, as unstratified analyses showed similar results, supporting our conclusions (data not shown).

Although a residual effect of follow-up length within each stratum cannot be excluded, we do not think this is very plausible, as recent evidence suggests that the improvement in HRQoL after completion of the initial rehabilitation-period is sustained up to 7 years after joint replacement surgery.[37], [38] The minimum follow-up period is well beyond the length of the expected rehabilitation-period, suggested by a recently published systematic review.[39]

Two other studies have investigated the relation between SEP and patient-reported outcomes after THR or TKR.[40], [41] Allen Butler et. al. have studied this relation in a randomised controlled trial, which compared two THR designs.[40] In this study, the effect of SEP was studied on a multitude of outcome measures, including the WOMAC, Short Form-12 (SF12) and degree of patient satisfaction. An association was found between lower levels of education and a degree of satisfaction which was “less than very satisfied”. Unfortunately, the authors have only reported their significant findings; differences in WOMAC or SF12 between social classes are not reported. Additionally, only p-values are reported instead of mean differences or relative risks, precluding any judgment on the clinical relevance of their findings. Finally, it is unclear for which factors any associations were adjusted, as the authors applied forward stepwise logistic regression modeling, without mentioning which variables were included in the final model. Davis et. al. have measured WOMAC scores before surgery and at 3, 12 and 24 months after TKR.[41] Whilst comparing WOMAC scores at each time point between patients of different income categories, patients with more disadvantaged SEP had worse preoperative WOMAC scores and similar postoperative WOMAC scores as patients with less disadvantaged SEP. These findings imply a larger improvement in disease-specific quality of life in patients with more disadvantaged SEP than in patients with less disadvantaged SEP. However, not all patients were measured at each time point. A cross-sectional comparison at each time point precludes judgment on the actual within-patient improvement in disease-specific quality of life. Due to methodological shortcomings of both other studies which investigated the relation between SEP and patient-reported outcomes after joint replacement, no meaningful comparison of results can be made.

Our findings have large implications for policymakers, as a more advantaged SEP is associated with greater use of health services in general.[12] A recent systematic review and numerous studies indicate that this also holds for THR[42]–[51] and TKR[42], [46]–[48], [50]–[52] in post-industrialised countries. Additionally, the need for joint replacement appears to be higher in patients with more disadvantaged SEP,[48], [51], [52] thereby increasing the inequity in access to joint replacement. Under-treatment of patients with more disadvantaged SEP cannot be justified, given the similar improvement in HRQoL and postoperative level of satisfaction with surgery between the examined groups of completed level of schooling.

A number of factors might explain the found differences in improvement in HRQoL after between THR and TKR patients per completed level of schooling groups. Biomechanical factors might play a role. The hip joint is a relatively simple ball and socket joint, which is adequately mimicked by a THR. The adequate mimicry of the biomechanics is reflected in a highly consistent improvement in HRQoL, regardless of completed level of schooling. The biomechanical aspects of the knee joint are more difficult to imitate, as the knee is a pivotal hinge joint with 6 degrees of freedom. These degrees of freedom are generally not restored after TKR, which is substantiated in kinematic and kinetic studies.[53] However, more complex biomechanics might explain a less consistent improvement in HRQoL in TKR patients, but does not explain differences between patient groups with different completed levels of schooling.

Differences between THR and TKR patients might be part of the explanation. Better general health, physical, emotional and social function, motivation and self-efficacy and lower levels of pain before surgery and during the rehabilitation period are associated with improved short- and medium-term outcomes.[2] In our study population, differences in the preoperative health status between completed level of schooling groups are more pronounced in TKR patients than in THR patients (table 2 and table 3). Finally, differences in rehabilitational options could play an important role. TKR patients require more rehabilitation than THR patients in order to achieve optimal results.[54] TKR patients with higher completed Level of Schooling might have better access to physical therapy or other rehabilitational facilities, and therefore gain more in role-physical functioning and general health than less advantaged patients. This effect might be exacerbated by the higher prevalence of obesity and co-morbidity in TKR patients compared to THR patients. Unfortunately, we do not have any information on the rehabilitational regime of our THR and TKR patients, leaving this hypothesis to be addressed in future research.

**Table 3:** Quality of Life before Knee Replacement: A Comparison Between Patients with different Completed Levels of Schooling.

SF36 Sub-Scale	University, Higher Vocational Education and Preparatory Higher Vocational & Scientific Education: n = 42	Middle Vocational Education and Preparatory Middle Vocational Education n = 98	Lower Vocational Education, Elementary Schooling and No Formal Education n = 122	All Patients: n = 262
Physical Functioning	40.4 (19.4)	41.3 (19.1)	38.4 (22.1)	39.8 (20.6)
Role-Physical	41.7 (41.9)	40.4 (42.3)	38.1 (42.9)	39.5 (42.4)
Bodily Pain	45.5 (19.4)	45.4 (19.7)	42.2 (21.6)	43.9 (20.6)
General Health	62.5 (19.0)	65.2 (18.7)	59.0 (21.1)	61.9 (20.0)
Vitality	63.2 (18.0)	63.1 (21.2)	57.7 (22.3)	60.6 (21.4)
Social Functioning	72.6 (22.1)	72.2 (23.0)	67.3 (26.3)	70.0 (24.5)
Role Emotional	82.5 (33.1)	74.5 (39.4)	62.0 (44.8)	70.0 (41.8)
Mental Health	79.6 (10.4)	76.4 (15.7)	68.1 (20.3)	73.1 (18.0)
PCS	36.8 (11.8)	40.2 (9.20)	40.8 (9.70)	39.9 (10.0)
MCS	55.5 (7.40)	53.0 (9.50)	48.8 (11.2)	51.5 (10.4)

## Supporting Information

Appendix S1 Scatterplots of the improvement in HRQoL as a function of the follow-up period length, stratified per completed levels of schooling, for Total Hip Replacement Patients. U+HVE+PHVSE: University, Higher Vocational Education and Preparatory Higher Vocational & Scientific Education; MVE+PMVE: Middle Vocational Education and Preparatory Middle Vocational Education; LVE+ES+NFE: Lower Vocational Education, Elementary Schooling and No Formal Education.
(PDF)

Appendix S2 Scatterplots of the improvement in HRQoL as a function of the follow-up period length, stratified per completed levels of schooling, for Total Knee Replacement Patients. U+HVE+PHVSE: University, Higher Vocational Education and Preparatory Higher Vocational & Scientific Education; MVE+PMVE: Middle Vocational Education and Preparatory Middle Vocational Education; LVE+ES+NFE: Lower Vocational Education, Elementary Schooling and No Formal Education.
(PDF)

Appendix S3 Scatterplots of the NRSS after surgery as a function of the follow-up period length, stratified per completed levels of schooling, for Total Hip Replacement Patients. U+HVE+PHVSE: University, Higher Vocational Education and Preparatory Higher Vocational & Scientific Education; MVE+PMVE: Middle Vocational Education and Preparatory Middle Vocational Education; LVE+ES+NFE: Lower Vocational Education, Elementary Schooling and No Formal Education.
(PDF)

Appendix S4 Scatterplots of the NRSS after surgery as a function of the follow-up period length, stratified per completed levels of schooling, for Total Knee Replacement Patients. U+HVE+PHVSE: University, Higher Vocational Education and Preparatory Higher Vocational & Scientific Education; MVE+PMVE: Middle Vocational Education and Preparatory Middle Vocational Education; LVE+ES+NFE: Lower Vocational Education, Elementary Schooling and No Formal Education.
(PDF)
