# Supplementary material for: Socio-Economic Position Has No Effect on Improvement in Health-Related Quality of Life and Patient Satisfaction in Total Hip and Knee Replacement: A Cohort Study
Source: PLoS One. 2013 Mar 8;8(3):e56785. doi: 10.1371/journal.pone.0056785 (PMC3592876; doi:10.1371/journal.pone.0056785)
Supplement: Appendix S1 — Scatterplots of the improvement in HRQoL as a function of the follow-up period length, stratified per completed levels of schooling, for Total Hip Replacement Patients. U+HVE+PHVSE: University, Higher Vocational Education and Preparatory Higher Vocational & Scientific Education; MVE+PMVE: Middle Vocational Education and Preparatory Middle Vocational Education; LVE+ES+NFE: Lower Vocational Education, Elementary Schooling and No Formal Education. (PDF) [file pone.0056785.s001.pdf]

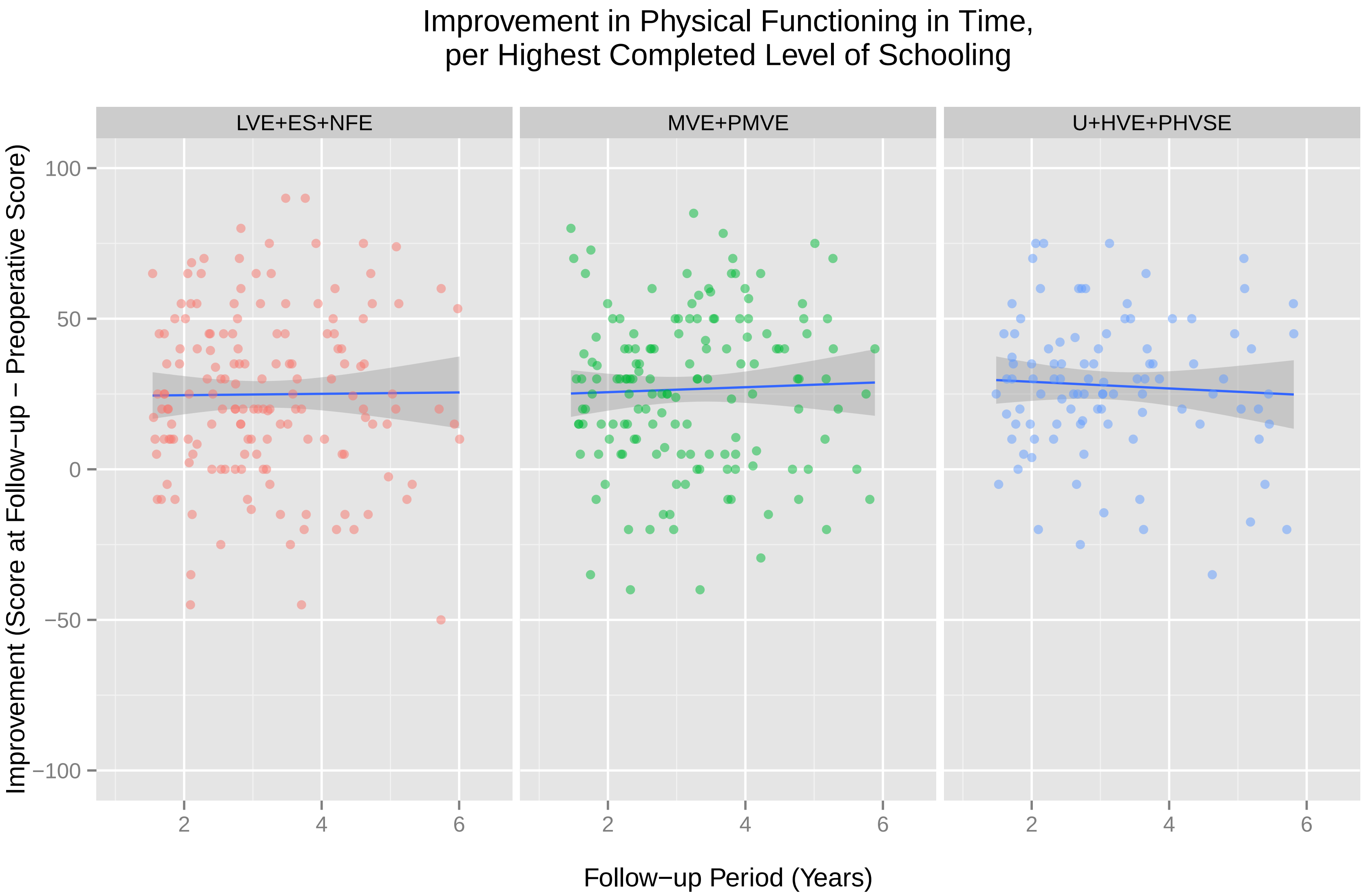

Improvement in Physical Functioning in Time,
per Highest Completed Level of Schooling
LVE+ES+NFE
MVE+PMVE
U+HVE+PHVSE
Improvement (Score at Follow−up − Preoperative Score)
100
50
0
−50
−100
2
4
6
Follow−up Period (Years)

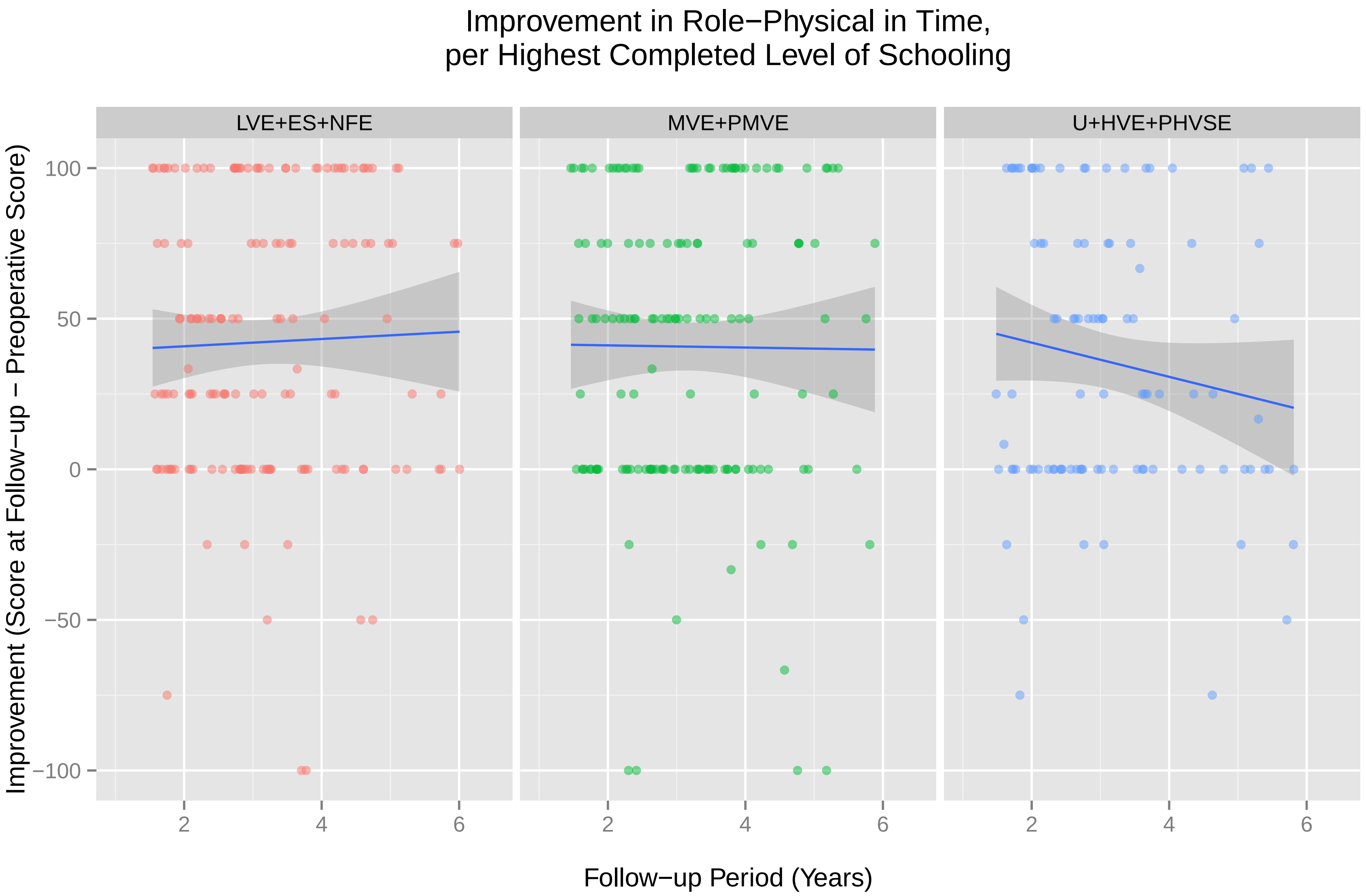
Improvement in Role−Physical in Time,
per Highest Completed Level of Schooling
LVE+ES+NFE
MVE+PMVE
U+HVE+PHVSE
Improvement (Score at Follow−up − Preoperative Score)
100
50
0
−50
−100
2
4
6
Follow−up Period (Years)

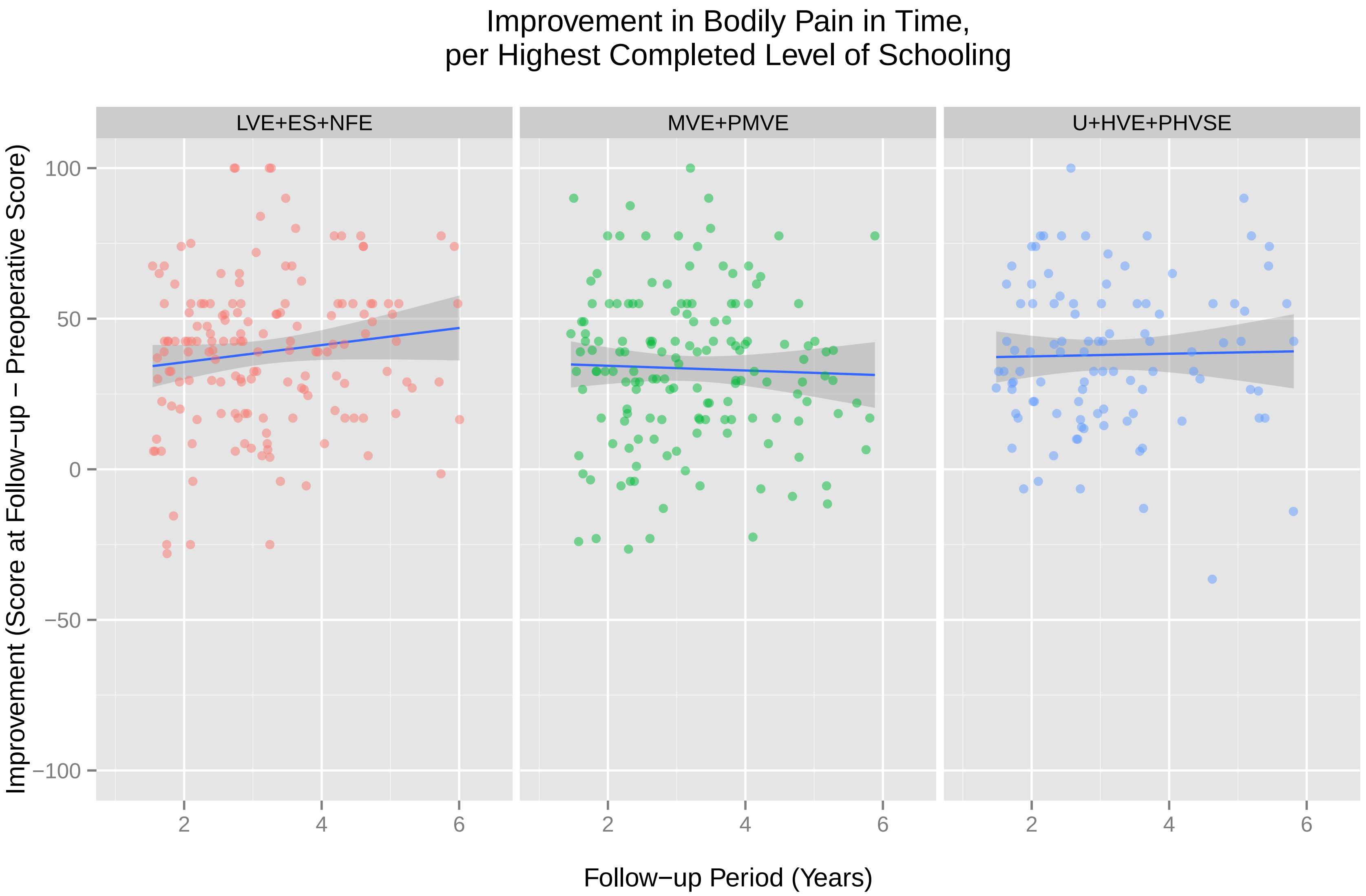

Improvement in Bodily Pain in Time,
per Highest Completed Level of Schooling
LVE+ES+NFE
MVE+PMVE
U+HVE+PHVSE
100
50
0
−50
−100
2
4
6
Improvement (Score at Follow−up − Preoperative Score)
Follow−up Period (Years)

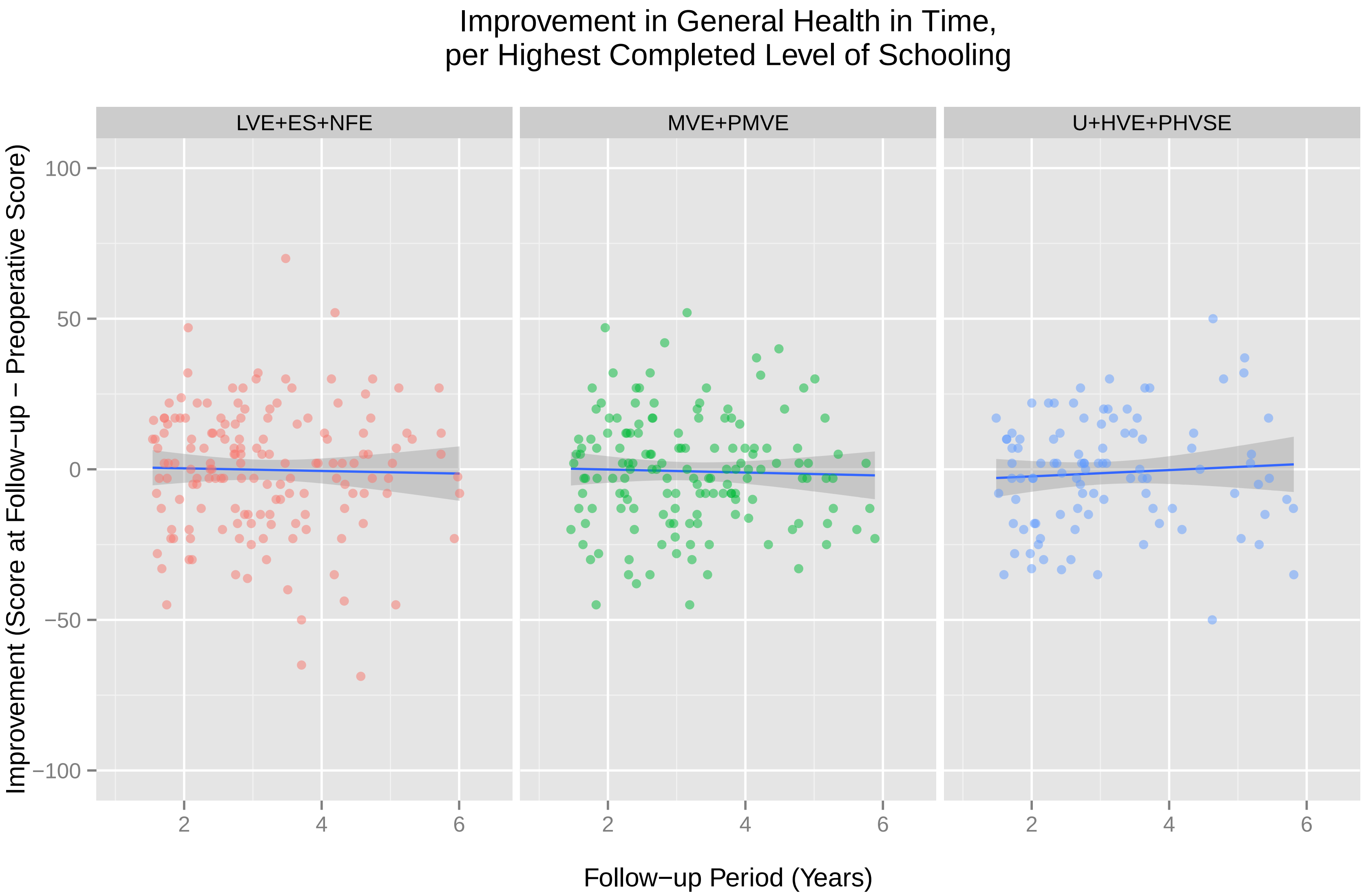
Improvement in General Health in Time,
per Highest Completed Level of Schooling
LVE+ES+NFE
MVE+PMVE
U+HVE+PHVSE
Improvement (Score at Follow−up − Preoperative Score)
100
50
0
−50
−100
2
4
6
Follow−up Period (Years)

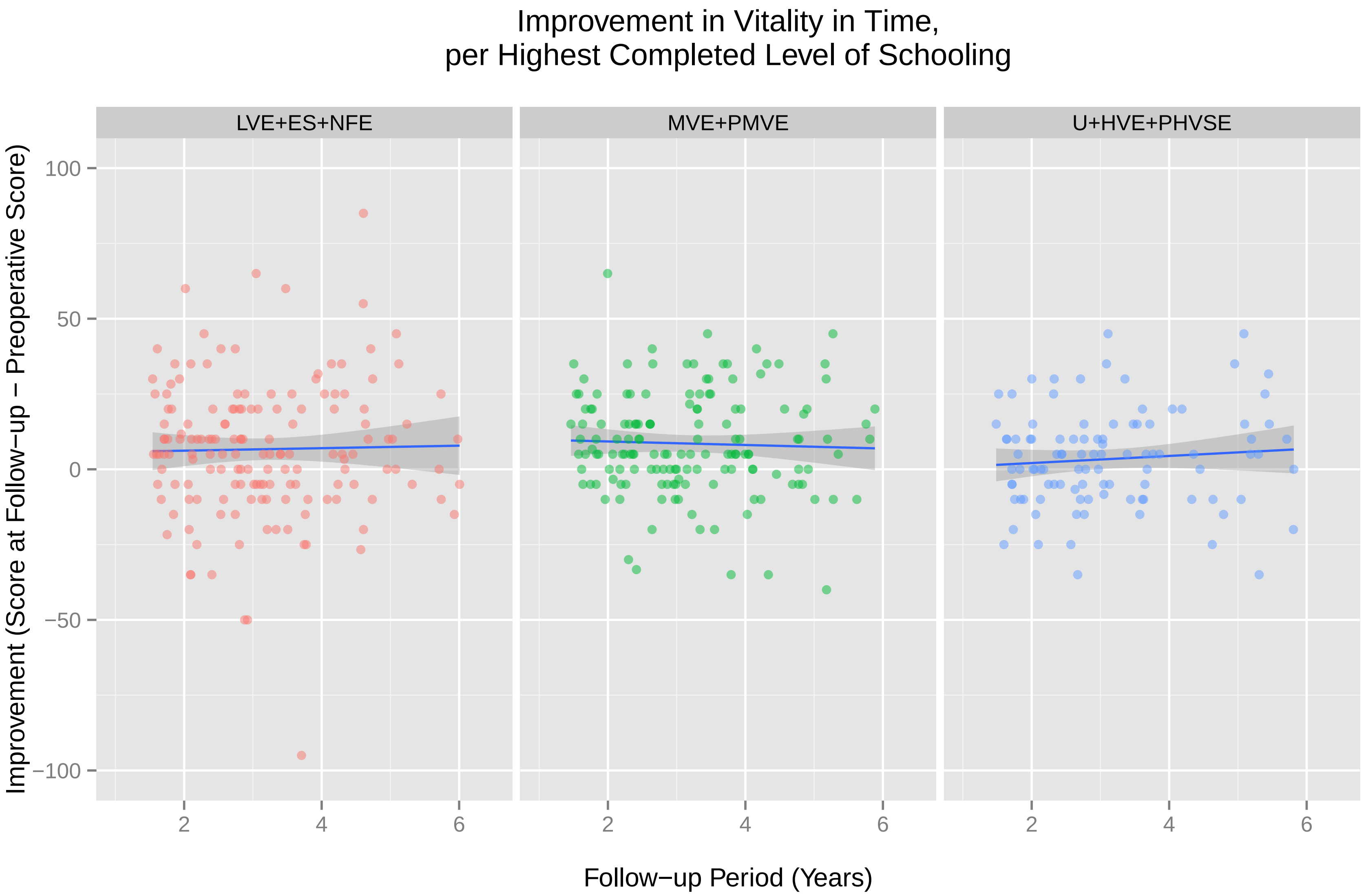

Improvement in Vitality in Time,
per Highest Completed Level of Schooling
LVE+ES+NFE
MVE+PMVE
U+HVE+PHVSE
Improvement (Score at Follow−up − Preoperative Score)
100
50
0
−50
−100
2
4
6
Follow−up Period (Years)

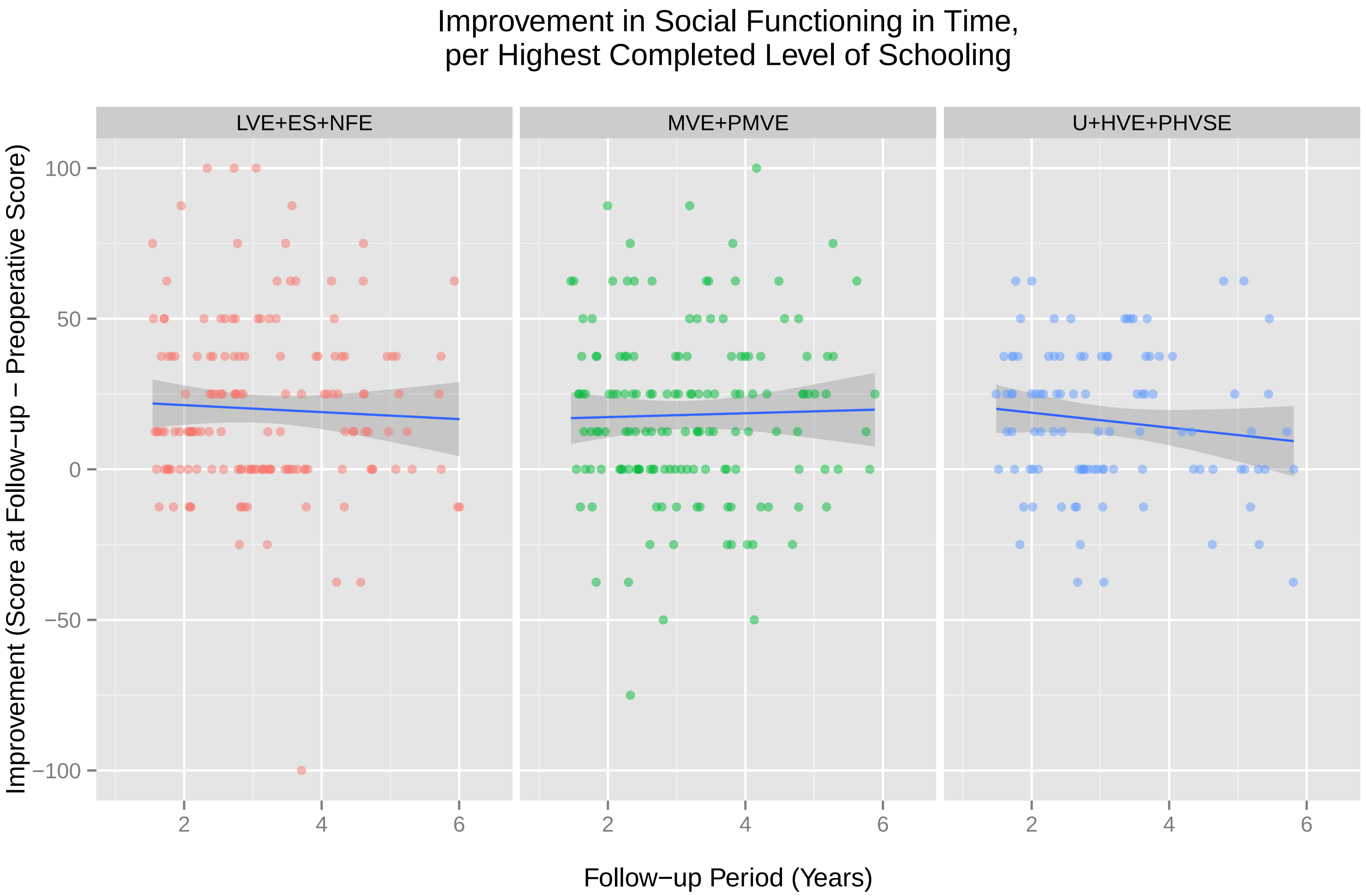
Improvement in Social Functioning in Time,
per Highest Completed Level of Schooling
LVE+ES+NFE
MVE+PMVE
U+HVE+PHVSE
Improvement (Score at Follow−up − Preoperative Score)
100
50
0
−50
−100
2
4
6
Follow−up Period (Years)

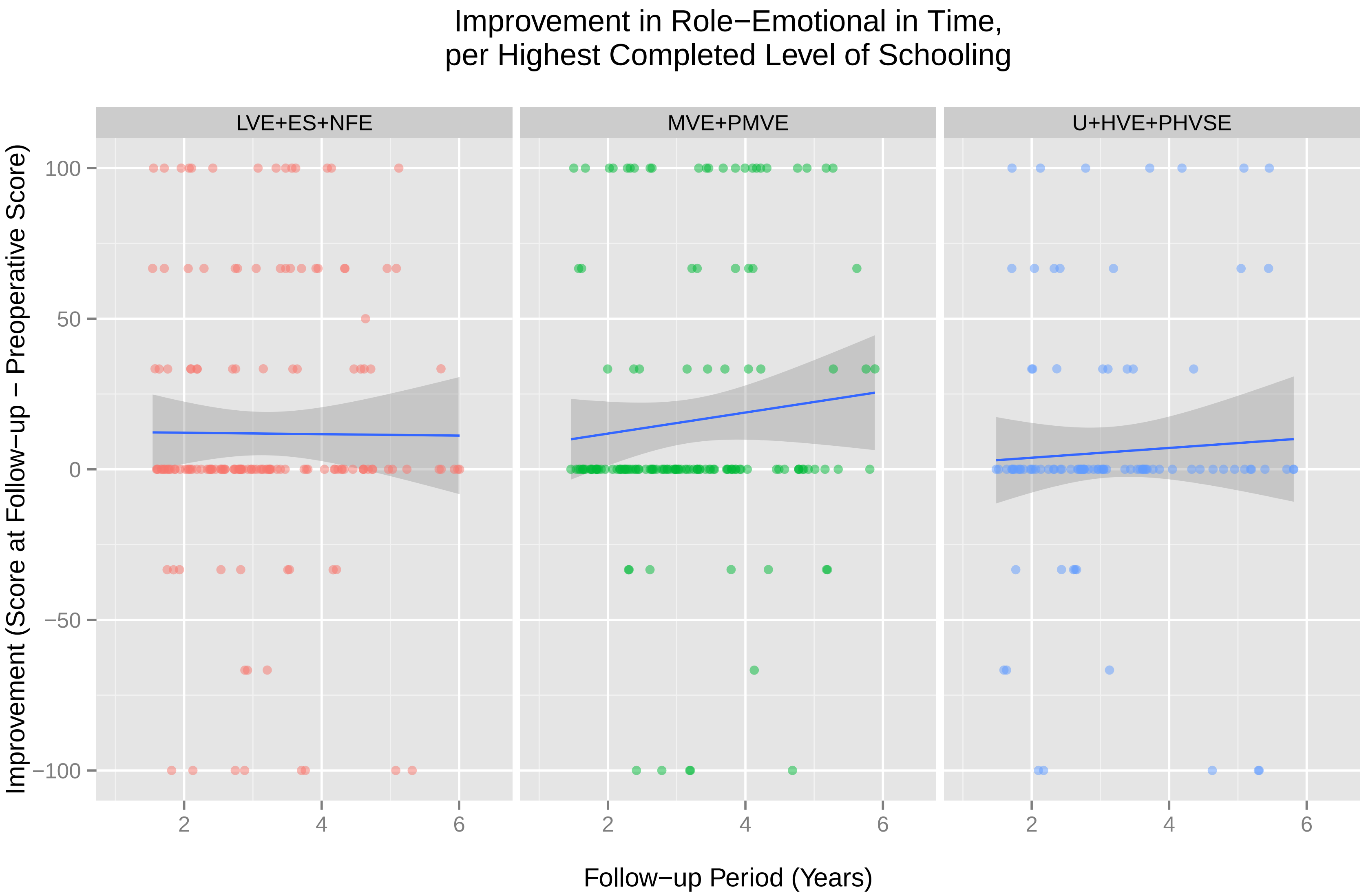
Improvement in Role−Emotional in Time,
per Highest Completed Level of Schooling
LVE+ES+NFE
MVE+PMVE
U+HVE+PHVSE
Improvement (Score at Follow−up − Preoperative Score)
100
50
0
−50
−100
2
4
6
Follow−up Period (Years)

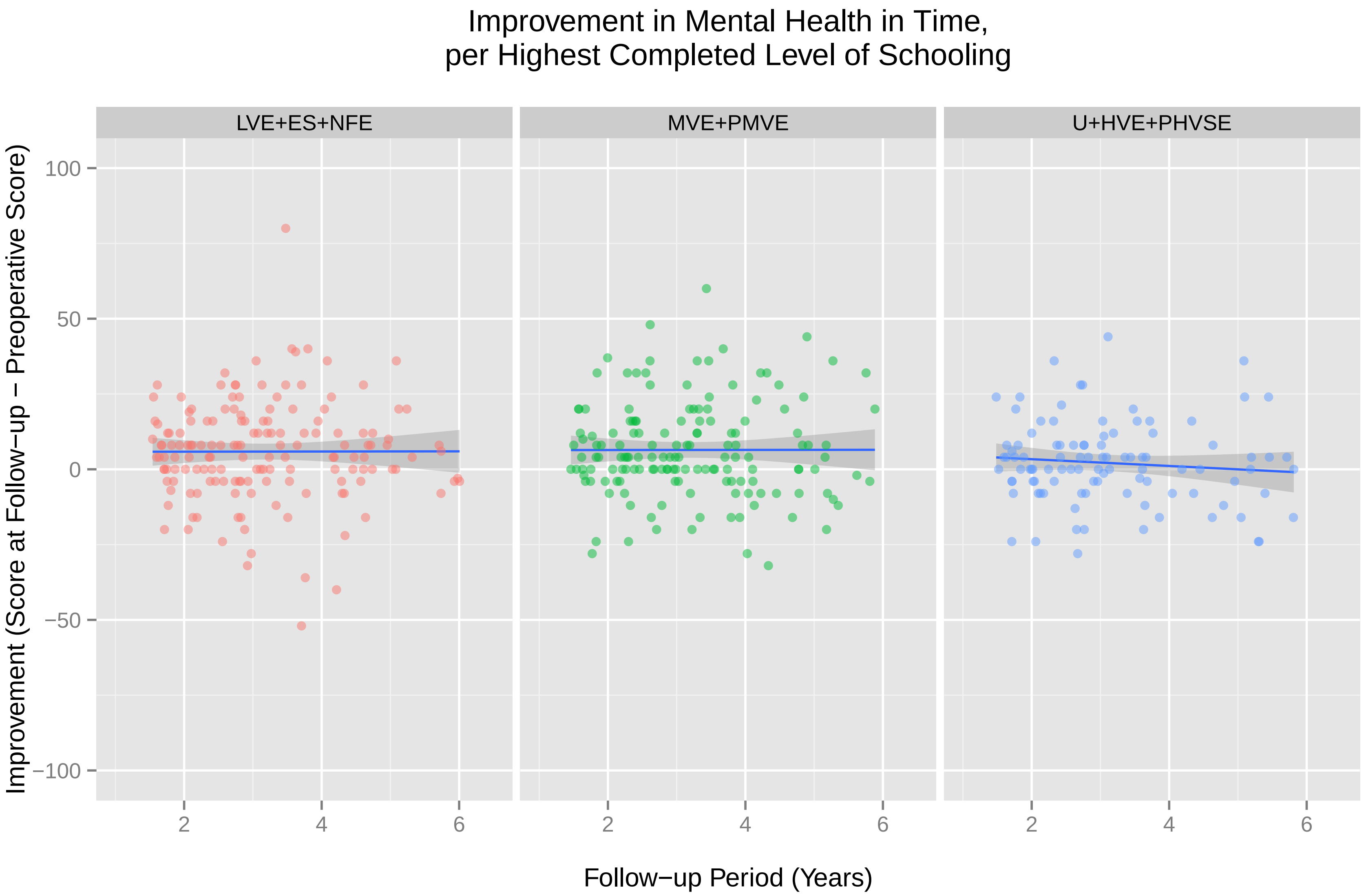

Improvement in Mental Health in Time,
per Highest Completed Level of Schooling
LVE+ES+NFE
MVE+PMVE
U+HVE+PHVSE
Improvement (Score at Follow−up − Preoperative Score)
100
50
0
−50
−100
2
4
6
Follow−up Period (Years)

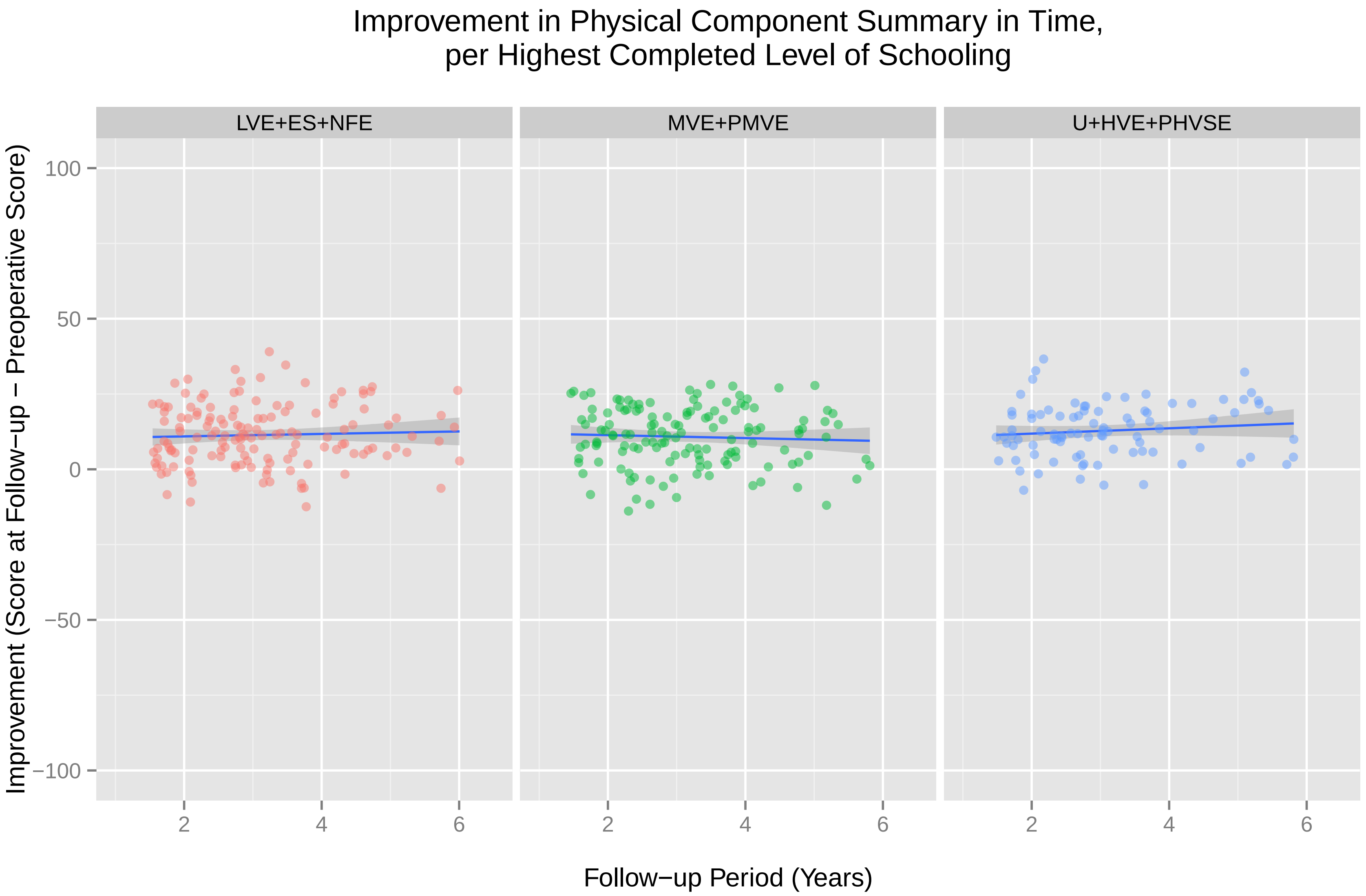

Improvement in Physical Component Summary in Time,
per Highest Completed Level of Schooling
LVE+ES+NFE
MVE+PMVE
U+HVE+PHVSE
Improvement (Score at Follow−up − Preoperative Score)
100
50
0
−50
−100
2
4
6
Follow−up Period (Years)

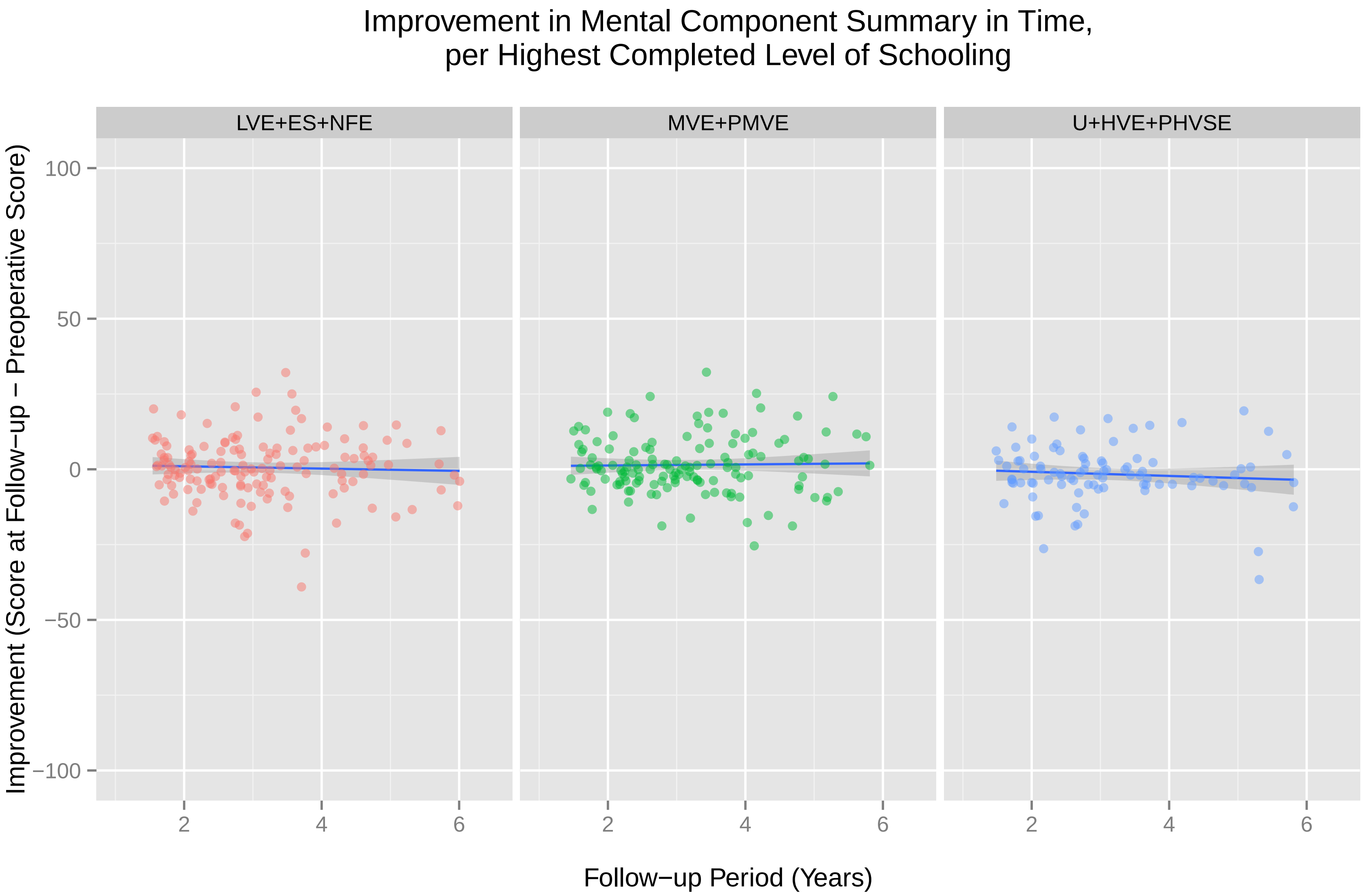
Improvement in Mental Component Summary in Time,
per Highest Completed Level of Schooling
LVE+ES+NFE
MVE+PMVE
U+HVE+PHVSE
100
50
0
−50
−100
2
4
6
Improvement (Score at Follow−up − Preoperative Score)
Follow−up Period (Years)
