# Supplementary figures and images for: Socio-Economic Position Has No Effect on Improvement in Health-Related Quality of Life and Patient Satisfaction in Total Hip and Knee Replacement: A Cohort Study
Source: PLoS One. 2013 Mar 8;8(3):e56785. doi: 10.1371/journal.pone.0056785 (PMC3592876; doi:10.1371/journal.pone.0056785)

# Satisfaction with Surgical Results, per Highest Completed Level of Schooling

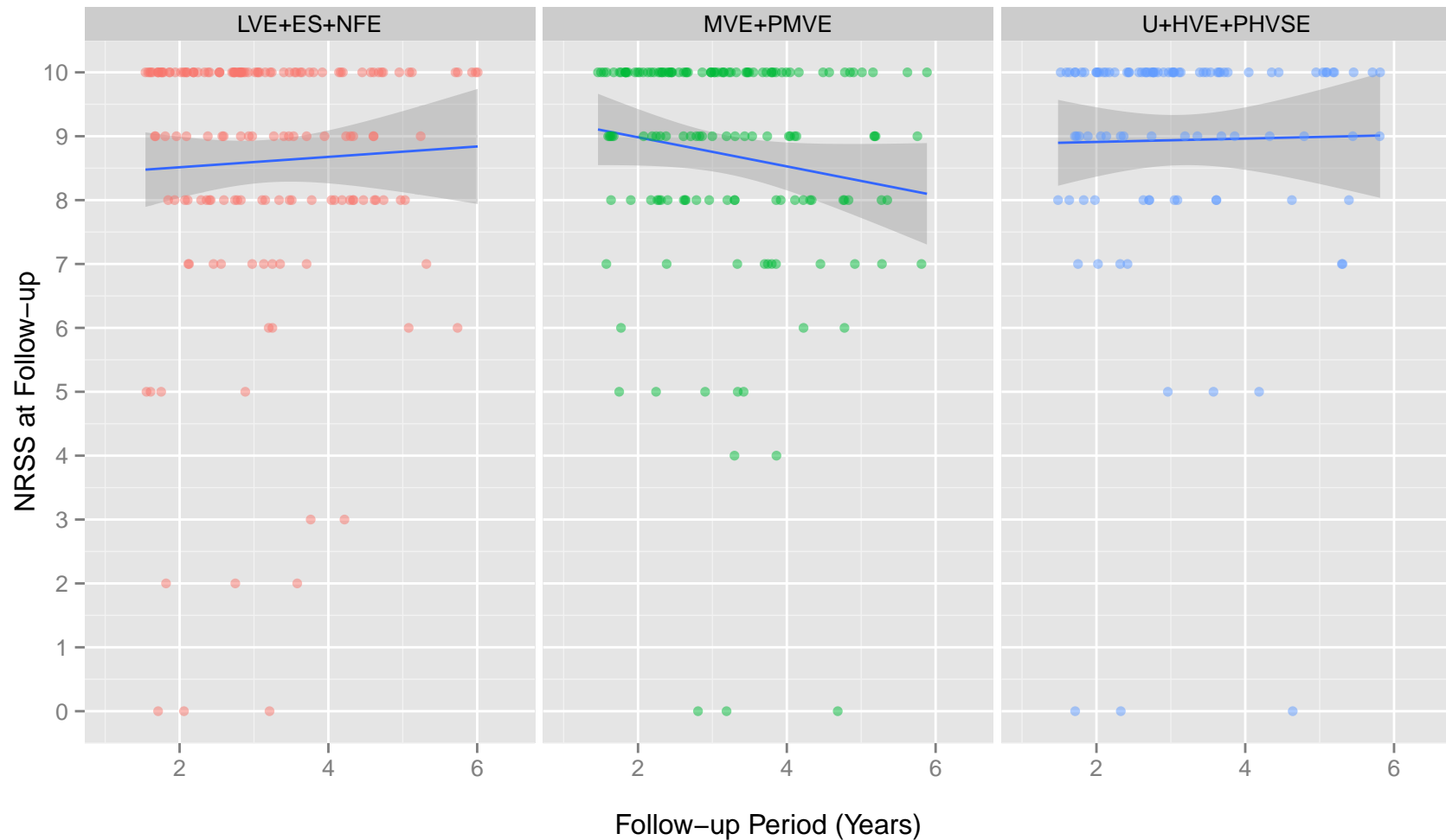

Supplement: Appendix S3 — Scatterplots of the NRSS after surgery as a function of the follow-up period length, stratified per completed levels of schooling, for Total Hip Replacement Patients. U+HVE+PHVSE: University, Higher Vocational Education and Preparatory Higher Vocational & Scientific Education; MVE+PMVE: Middle Vocational Education and Preparatory Middle Vocational Education; LVE+ES+NFE: Lower Vocational Education, Elementary Schooling and No Formal Education. (PDF) [file pone.0056785.s003.pdf]

# Satisfaction with Surgical Results, per Highest Completed Level of Schooling

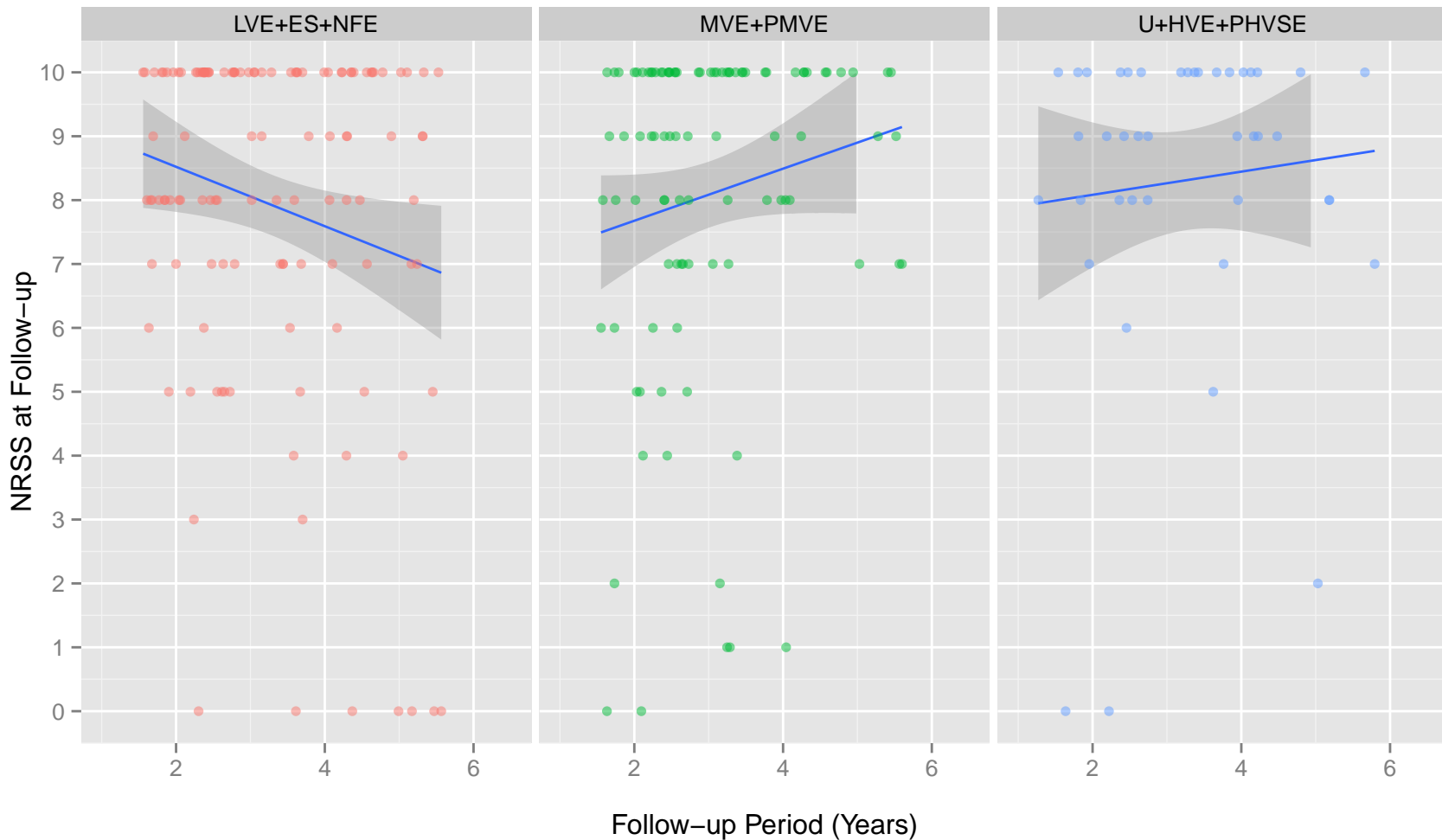

Supplement: Appendix S4 — Scatterplots of the NRSS after surgery as a function of the follow-up period length, stratified per completed levels of schooling, for Total Knee Replacement Patients. U+HVE+PHVSE: University, Higher Vocational Education and Preparatory Higher Vocational & Scientific Education; MVE+PMVE: Middle Vocational Education and Preparatory Middle Vocational Education; LVE+ES+NFE: Lower Vocational Education, Elementary Schooling and No Formal Education. (PDF) [file pone.0056785.s004.pdf]
